# Path Planning for 3-D In-Hand Manipulation of Micro-Objects Using Rotation Decomposition

**DOI:** 10.3390/mi12080986

**Published:** 2021-08-19

**Authors:** Pardeep Kumar, Michaël Gauthier, Redwan Dahmouche

**Affiliations:** FEMTO-ST Institute, Université Bourgogne Franche-Comté CNRS, 25000 Besançon, France; michael.gauthier@femto-st.fr (M.G.); redwan.dahmouche@femto-st.fr (R.D.)

**Keywords:** micro-manipulation, micro-handling, finger path planning

## Abstract

Robotic manipulation and assembly of micro and nanocomponents in confined spaces is still a challenge. Indeed, the current proposed solutions that are highly inspired by classical industrial robotics are not currently able to combine precision, compactness, dexterity, and high blocking forces. In a previous work, we proposed 2-D in-hand robotic dexterous manipulation methods of arbitrary shaped objects that considered adhesion forces that exist at the micro and nanoscales. Direct extension of the proposed method to 3-D would involve an exponential increase in complexity. In this paper, we propose an approach that allows to plan for 3-D dexterous in-hand manipulation with a moderate increase in complexity. The main idea is to decompose any 3-D motion into a 3-D translation and three rotations about specific axes related to the object. The obtained simulation results show that 3-D in-hand dexterous micro-manipulation of arbitrary objects in presence of adhesion forces can be planned in just few seconds.

## 1. Introduction

Micro-manipulation has been an active field of research for more than three decades [1,2]. We can distinguish two approaches to manipulate micro-objects. The first one is non-contact manipulation, where the forces (magnetic or electrostatic, for instance) are exerted at a certain distance from the manipulated objects [3,4]. This approach has many benefits such as direct action on specific objects (cells, etc.) from a certain distance, a short response time, and the capacity of using highly miniaturized objects as manipulators (particles, flagellates, etc.) However, non-contact manipulation suffers from some drawbacks such as low blocking forces, the sensitivity to the used material, and the fact that the produced forces are not localized, which might affect the other objects in the workspace. The other approach is contact manipulation, which is able to compensate for some of these drawbacks.

The most used physical architecture in micro-manipulation consists of a simple tweezers mounted on a serial or parallel robot [5,6]. This architecture is inspired by classical industrial robots and is not necessarily the most adequate for micro-manipulation. Indeed, the ratio between the robot’s size and manipulated object is much higher in micro-manipulation. For example, let us consider a compact robot of 10 cm side manipulating a 10 μm cell. The ratio between the robot dimension and cell is about $10^{4}$. From the size ratio point of view, it is like manipulating a 10 cm side object with a robot which is 1 km large. We can thus see that any relative uncertainty in the mechanical structure of the robot has a huge impact on the precision and accuracy of the robot and the reliability of the performed task. In addition, any rotation of the object would require the rotation of the whole robot, which is unsuitable when manipulating in confined spaces such as inside Scanning Electron Microscopes [7] or inside the human body [8].

One way to avoid those issues is to perform local rotations through dexterous in-hand manipulation. Robotic dexterous manipulation has been an active field of research for more than three decades [9,10,11]. Most research in this field is focused on grasping and manipulation of daily life objects using anthropomorphic hands [12,13]. The objective is to allow mobile manipulators and humanoid robots to manipulate arbitrary shaped objects typically made for humans. The problem consists in finding of the successive stable grasps able to reconfigure the position and orientation of the manipulated object. Several strategies are used to this end, such as rolling without sliding, finger gaiting, and others [14].

The problem of in-hand manipulation of arbitrarily shaped objects is very complex. In [15], a huge computation power composed of 384 workers (each worker has 16 core CPU) and 8 GPUs (NVIDIA V100) for policy training which results in the generation of 2 years of simulated experience per hour and a system with 32 CPU cores and 3 GPUs (NVIDIA P40) for vision model training were used to train the system to perform low accuracy manipulation operation using an anthropomorphic hand.

In industrial manipulation in general and micro-manipulation in particular, the problem of dexterous in-hand manipulation can be somehow simplified. Indeed, robot architecture and the shapes of the manipulated objects are usually known before manipulation, since they are parts of the manufacturing process. This allows us to perform pre-computation operations that are invariant with regard to the manipulating robot and properties of the manipulated object [16].

In micro-manipulation, the kinematics for each probe (or finger) of the manipulating robot is usually simple, such as Cartesian architectures with pure translations are common [17]. Those differences might help to reduce the complexity of dexterous in-hand manipulation, which also highly depends on the considered shapes and the number of possible contact points *c*. Considering simple polyhedrons such as cuboids [1,18], tetrahedrons, or objects with a limited number of facets has already been resolved [19,20].

However, when the complexity of the shape increases because of curved surfaces, the problem becomes much more complex. In [16,21,22], automatic 2-DoF manipulation of arbitrary shaped objects was accomplished by pre-computing a graph that represents all the stable grasps and the possible operations (rolling without sliding, finger gaiting, etc.) The proposed method was also able to take into account the adhesion forces that, on the one hand, improves the stability of the grasps but, on the other hand, disturbs the grasp when detaching one or several fingers. Extending those approaches from 2-D to 3-D induces a significant increase in complexity. Indeed, the number of potential contact points *c* in 3-D (on object surface) is much higher than in 2-D (on curve). In addition, the number of DoF (3 to 6) as well as the number of fingers required for grasping and manipulating increases. All those parameters make the grasp space significantly bigger and the manipulation problem much more complex.

The contribution of this paper is thus to propose a 3-D dexterous in-hand micro-manipulation approach from a series of 2-D manipulation steps. Indeed, considering that all the fingers can only translate as it is the case in most multi-contact micro/nano-manipulation platforms; translating any object consists in synchronously translating all the fingers. When it comes to rotations, we know that any 3-D rotation can be decomposed into 3 successive rotations about 2 different axes in the current frame (XYX rotations, for instance). One can thus define two axes on the object to be considered for rotations. The normal plans to the two rotation axes intersect with the surface of the manipulated object and define two 2-D curves. Finally, rotating the object in 3-D consists in rotating it successively in 2-D considering the defined curves. The previously developed methods can thus be considered for 3-D manipulation. However, some key differences when switching from one rotation step to another must be considered to guarantee the continuity of the manipulation. These differences lead to new constraints when planning the manipulation that must be considered when formalizing the manipulation problem and proposing a solution for it.

In other words, this paper provides the first method to define 3-D finger trajectories enabling to perform in-hand 3-D rotations of a micro-object. The method is able to take into account some of the microscale specificities such as the adhesion.

The next section introduces the physical modeling for micro-manipulation in presence of adhesion forces. Section 3 formalizes the problem of the complexity increase and the proposed approach to leverage its effect. The manipulation strategy based on a graph search algorithm is then presented in Section 4. The simulation results are presented in Section 5 followed by a discussion and conclusion section.

## 2. Modeling and Background

This section formalizes the grasping and in-hand manipulation problem at micro-scale.

### 2.1. Grasping Forces

Let us consider *F* fingers grasping an object. As proposed in [23], we assume in micro-scale that the contact forces can be modelled using a combination of the *Coulomb’s Law* in which the friction magnitude is independent of the velocity and contact area and the *pull-off force* ($f_{po}$) representing the force required to detach the finger from the object as:

(1) $$\sqrt{f_{x}^{2}+f_{y}^{2}}\leq \mu \left(f_{n}+f_{po}\right)\;,$$

where $f_{x}$ and $f_{y}$ are the tangential components of the force on *x*-axis and *y*-axis respectively, $f_{n}$ is the normal component of force, $f_{po}$ is pull-off force, and μ is the friction coefficient.

Due to the presence of the *pull-off force* ($f_{po}$), it is possible to apply a negative force (pulling the object) in micro-scale since the applied force lies in the modified friction cone, whereas in macro-scale, only the positive grasping forces (pushing the object) are possible.

### 2.2. Grasping Equilibrium

The manipulated object must be in equilibrium during the whole manipulation process. The equilibrium condition for a rigid body is that the sum of all the wrenches should be equal to zero, whereas, a wrench vector is composed of forces and torques at a contact point as provided by

(2) $$w=\left[\begin{array}{c} f\\ \tau \end{array}\right],$$

where *w* is a wrench, and *f* and τ represent the force and torque, respectively. A grasp using *F* fingers is stable if

(3) $$\sum_{i=1}^{F}w_{i}+w_{ext}=0\;,$$

where $w_{i}$ is the grasping wrench applied by *i*th finger, and $w_{ext}$ is the external wrench applied to the object.

### 2.3. Impact of Pull-Off Forces on Finger Gaiting

Pull-off force has an important role in re-grasping and finger gaiting (i.e., fingers replacement). When a finger (e.g., the Fth finger) is being detached, it will pull the object with a force corresponding to the pull-off force ($f_{po}$). This pull-off force may disturb the grasping equilibrium. Thus, it is necessary for the remaining F−1 fingers to compensate for this $f_{po}$ to maintain the object’s stability. Thus, removing the Fth finger is possible only if the following equation is satisfied:

(4) $$\sum_{i=1}^{F-1}w_{i}+w_{ext}+w_{po}=0\;,$$

where $w_{po}$ is the pull-off wrench caused by releasing the Fth finger.

## 3. Problem Formalization

In order to perform fingers’ path planning, we assume that the object’s shape is known through its Computer-Aided Design model (CAD model). Considering the small size of the manipulated object, we also assume that the fingers are placed on their respective 3-D translation stages as it is widely done in robotic micromanipulation (e.g., in [24]) and that the translation ranges are significantly larger than the object’s dimensions. We also consider only two and three fingers grasps. The admissible contact points (*c*) on the object are obtained by sampling the object’s surface (see Section 3.2).

To generate finger trajectories, we build a Graph(G), where each stable grasp is connected to its subsequent stable grasp(s). Each element of the graph is considered as a Node(n) which is connected by Edges(e) to other nodes. Finding an optimal finger trajectory to rotate an object consists in finding a path in the Graph(G). Each Node(n) of the Graph is represented by four parameters, i.e. n=[ijkl] where *i*, *j*, and *k* define the indexes of contact points on the object of the first, second and third fingers respectively, and *l* defines the index of angular position of the object. Considering that the index of a finger is 0 when the finger is not grasping the object, and *c* is the number of contact points, the indexes *i*, *j*, and *k* are integer between 0 and *c*. The Edges(e) represent the links between two subsequent stable grasps. It includes edges representing (i) rotation of the object taking into account the fingers’ rolling, (ii) a finger placement on the object, and (iii) a finger removal. The construction of the Graph(G) has been discussed in detail in [16]. Moreover, we also briefly describe the construction of the Graph in Section 3.3 for its better understanding.

For fingers’ path planning, we use $A^{*}$ algorithm whose computational complexity is $O(b^{d})$ where *b* is the branching factor (average number of branches per node), and *d* is the depth (number of nodes to reach the goal). For branching factor *b*, there are two cases, the first case is when two fingers are in use, and the second is when three fingers are used. For the first case, when two fingers are already in contact with the object, the branching factor is the sum of operations: clockwise rotation, counter-clockwise rotation, and addition of the third finger over the remaining c−2 contact points. Whereas for the second case, when three fingers are in use, the branching factor is the sum of operations: clockwise rotation, counter-clockwise rotation, and detaching one of the fingers. For depth *d*, the total number of nodes are all the possible equilibrium grasps, which is the result of the number of contact points *c* of an object, the total number of fingers (*F*) being used to manipulate the object, and the number of possible angular positions/orientations ($n_{\theta }$) of the object. The higher the number of nodes, the more time the algorithm will take to find the trajectory. Thus, we propose to reduce the depth using Euler’s angles in order to reduce the computational complexity.

### 3.1. Reducing the Complexity Using Euler’s Angle

In planar (2-D) manipulation, where we consider manipulating an object in a single plane, the depth *d* is computed as

(5) $$d_{2D}\approx c^{F}\cdot n_{\theta }\;.$$

Whereas to manipulate an object in spatial case (S), we imagine P number of planes over which the contact points are generated. In this case, the depth *d* is computed as

(6) $$d_{SD}\approx {\left(P\cdot c\right)}^{F}\cdot n_{\theta }=P^{F}\cdot d_{2D}\;.$$

**Figure 1:**
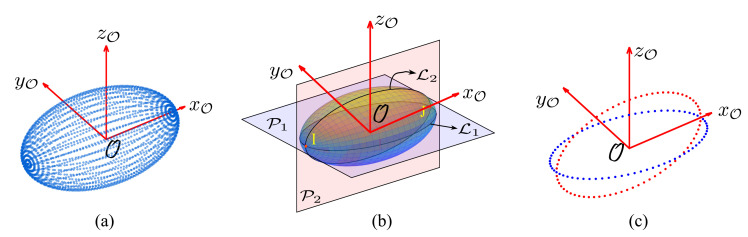
Illustration of complexity reduction; (**a**) contact points when P numbers of planes are considered, (**b**) introducing two orthogonal planes $P_{1}$ and $P_{2}$ resulting projection lines $L_{1}$ and $L_{2}$ over object, (**c**) contact points after introducing two orthogonal planes.

**Figure 2:**
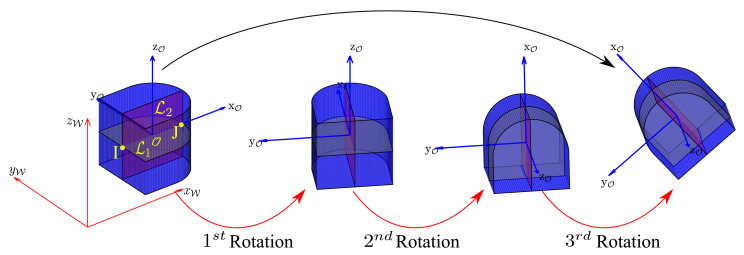
Example of a decomposition of 3-D rotation into three successive rotations, where red and blue reference frames represent world frame and object frame, respectively. The first rotation consists in $R(Z_{O},\theta_{1})$ over $P_{1}$, the second rotation is $R(Y_{O},\theta_{2})$ over $P_{2}$, and the third rotation is defined by $R(Z_{O},\theta_{3})$ over $P_{1}$.

From Equation (6), we can conclude that the increase in depth exponentially increases the computational complexity. One of the challenges in 3-D manipulation is to leverage the complexity of the search algorithm. According to *Euler’s Angles*, it is possible to decompose any 3-D rotation into three individual 2-D rotations along any two orthogonal axes [25]. Thus, we define a frame defining the object position as $\left(O,X_{O},Y_{O},Z_{O}\right)$ and propose to only retain two orthogonal planes $P_{1}$ at $X_{O}Y_{O}$-axis and $P_{2}$ at $X_{O}Z_{O}$-axis intersecting the object over lines $L_{1}$ and $L_{2}$ as illustrated in Figure 1b. Since, the three individual 2-D rotations will be carried out in two different planes, we consider the two intersecting points *I* and *J* of lines $L_{1}$ and $L_{2}$ as a common link for these three individual rotations. The three individual rotations will be carried out as: $R(Z_{O},\theta_{1})$ over $P_{1}$, $R(Y_{O},\theta_{2})$ over $P_{2}$, and $R(Z_{O},\theta_{3})$ over $P_{1}$, respectively, as represented in Figure 2, which will limit the branching factor and reduce the number of grasps to the sum of grasping possibilities of each rotation carried out over these two planes as

(7) $$d_{3D}=2\cdot d_{2D_{L_{1}}}+d_{2D_{L_{2}}}.$$

The reduction of complexity introduced by two planes also reduces the genericity of our approach. Indeed, the considered objects should have a geometry in which both planes $P_{1}$ and $P_{2}$ can be defined. However, this is usually the case for micro-objects manufactured using the classical microfabrication methods.

### 3.2. Sampling Strategy

As we consider that the object’s CAD model is known, we sample the manipulation lines $L_{1}$ and $L_{2}$ to generate contact points (*c*) for grasping. For sampling on the object, we consider that the rolling between two successive sample points corresponds to a pre-defined rotation Δθ of the object.

**Figure 3:**
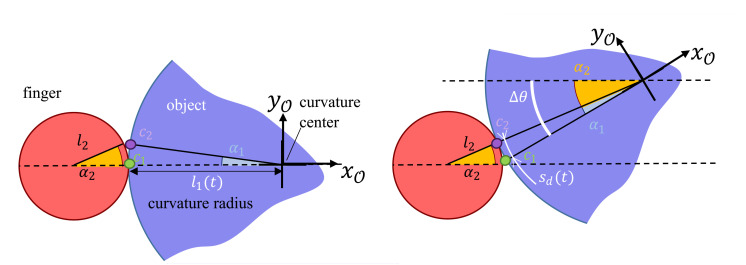
Representation of finger rotating an object to formulate the sampling formula.

In that case, the distance $s_{d}\left(t\right)$ between two contact points is the function of finger radius $l_{2}$, the rotational step Δθ, and the local curvature radius of the object $l_{1}\left(t\right)$. To formulate the equation, we will refer to Figure 3, where $c_{1}$ is the contact point from where the object starts rotating around the finger, and $c_{2}$ is the contact point where the rotation finishes. As the object’s total rotation (Δθ) is the sum of the two angles $\alpha_{1}$ and $\alpha_{2}$, and since both rolling distances $s_{d}\left(t\right)=\alpha_{2}\cdot l_{2}$ on the finger and $s_{d}\left(t\right)=\alpha_{1}\cdot l_{1}\left(t\right)$ on the object are equal, we can determine the instantaneous curvilinear sampling distance $s_{d}\left(t\right)$:

(8) $$s_{d}\left(t\right)=\frac{\left|\Delta \theta \right|\cdot l_{2}}{l_{1}\left(t\right)+l_{2}}\cdot l_{1}\left(t\right),$$

where the curvature radius of the object $l_{1}\left(t\right)$ on $X_{O}Y_{O}$-axis can be determined using the curvature formula defined for parametric equation:

(9) $$l_{1}\left(t\right)=1/\kappa =\frac{{\left({x^{\prime }\left(t\right)}^{2}+{y^{\prime }\left(t\right)}^{2}\right)}^{3/2}}{|x^{\prime }\left(t\right)y^{\prime \prime }\left(t\right)-y^{\prime }\left(t\right)x^{\prime \prime }\left(t\right)|},$$

where $x^{\prime }$, $y^{\prime }$, $x^{\prime \prime }$, and $y^{\prime \prime }$ are the first and second derivatives of the parametric equation.

### 3.3. Graph G

The sampling strategy (Equation (8)) enables to define the location and number of contact point along the lines lines $L_{1}$, $L_{2}$. Concretely, it means that we consider a discrete number (*c*) of contact points and discrete rotations defined by the constant rotation step Δθ. Parameters *i*, *j*, *k* are integers in [0;c] (0 means that the finger is not used in the grasp), and parameter *l* is integer in [1;1+360/Δθ] meaning that the initial position θ=0 is defined by l=1. As an example, n=[ijkl]=[0257] means that the first finger is not used (i=0), the second and third fingers are respectively placed on contact position 2 and 5 (j=2, k=5), and that the current angular position of the object defined by (l=7) is (7−1)·Δθ. The stability of each potential grasping is checked using the static equilibrium equation (Equation (3)). Each stable configuration becomes a Node(n) in the Graph(G), in which a path represents a manipulation sequence. The Nodes (*n*) are connected to the other to form the Graph (G) in the following way:

-  The 2-fingers grasps are respectively connected to (i) the 2-fingers grasp reachable by finger rolling following a clockwise rotation, (ii) the 2-fingers grasp reachable by finger rolling following a counter-clockwise rotation and (iii) to every 3-fingers grasps corresponding to a third finger addition.
-  The 3-fingers grasps are connected in a similar way to 3-fingers grasps (i) and (ii) and to (iii) three 2-fingers grasps considering each finger removing.

The next section defines the manipulation strategy including the cost and heuristic function to define an optimal path in the Graph(G), whose resolution using $A^{*}$ will be presented in Section 5.

## 4. In-Hand Manipulation Strategy

Given the number of fingers and object geometry, the finger trajectory generation is accomplished using two steps. The first step is to compute the stable grasps and generate the Graph (*G*) for each line $L_{1}$ and $L_{2}$, while the second step is to define a path by traversing the graph(s) to achieve the desired configuration.

The object’s modeling is restricted only to two lines $L_{1}$ and $L_{2}$ and intersecting contact points *I* and *J* being the only common link, induces some constraints for successive rotations. These constraints are:

- *The first rotation should end at the intersecting points I and J*.
- *The second rotation should start from these intersecting points I and J and end at the same intersecting points*.
- *The third rotation should start from these intersecting points I and J*.

In previous work, Seon et al. [16] achieved only desired pose of object for planar manipulation, while to manipulate the object in 3-D, we need to comply with these constraints. Thus, we propose an original method to provide finger trajectories using $A^{*}$ algorithm.

The $A^{*}$ algorithm uses a heuristic for traversing the graph(s), while ensuring that it computes a path with minimum cost through the nodes *n*. The algorithm optimizes the function f(n), which consists of the cost function g(n) and heuristic function h(n) as

(10) f(n)=g(n)+h(n).

Please note that in case of having two nodes with the same value of function f(n), we chose a classical “Tie breaking" strategy consisting in exploring first the node having the minimal heuristic h(n).

### 4.1. Cost Function g(n)

Cost function is the parameter which computes the cost of activity being performed to carry out the manipulation process from previous node ($n_{previous}$) to current node (*n*). As per our strategy, there are two operations that can be performed for the manipulation task; (i) rolling of object and (ii) reconfiguration of fingers (finger gaiting):

-  The “rolling operation”, changes the orientation of object when 2 or 3 fingers are used, reaching to node *n* from previous node $n_{previous}$ without any reconfiguration. Thus, we formalize the value of cost when a rolling operation is performed as
  (11) $$\begin{array}{c} g{\left(n\right)}_{rolling}=F_{used}\cdot \left|\Delta \theta \right|+g\left(n_{previous}\right), \end{array}$$
  where $F_{used}$ is the number of fingers used to roll the object, and $g(n_{previous})$ is the cost of the previous node.
-  When the “finger gaiting” operation is performed, either a finger is added or removed while the object remains in the same orientation; thus, the cost $g{\left(n\right)}_{gaiting}$ is formalized as
  (12) $$\begin{array}{c} g{\left(n\right)}_{gaiting}=g_{r}+g\left(n_{previous}\right), \end{array}$$
  where $g_{r}$ is the reconfiguration cost. Concretely, we define the value of reconfiguration cost $g_{r}$ at $|180^{\circ }|$. It means that we assume that a reconfiguration has a similar cost to a quarter-turn of rotation using two fingers ($2\;\times \;|90^{\circ }|$).

### 4.2. Heuristic Function h(n)

In search algorithms, the heuristic function h(n) estimates the minimum cost from the current node n=[ijkl] to the goal node $n_{g}=\left[i_{g}\;j_{g}\;k_{g}\;l_{g}\;\right]$. Please note that i=0, j=0 or k=0 means the first, second or third finger is respectively not used. In order to guarantee the convergence of the $A^{*}$ algorithm, the heuristic must underestimate the remaining cost. To develop the heuristic function, we take into account all the parameters of a node (number of fingers being used, contact point on object, and angular position of object) as

(13) $$h\left(n\right)=2\cdot abs\left(l-l_{g}\right)\cdot \left|\Delta \theta \right|+t_{r}\left(\left\{h_{i},h_{j},h_{k}\right\}\right)\cdot g_{r},$$

where $abs(l-l_{g})$ is the difference between current orientation of object and goal orientation of object, Δθ is the pre-defined rotational step, and $t_{r}$ is the function of the non-ordered set $\left\{h_{i},h_{j},h_{k}\right\}$ which provides the total number of estimated reconfigurations (addition/removal operation). Each element i.e., $h_{i}$, $h_{j}$ and $h_{k}$ provides one of the four conditions in set H={nu,ar,dp,ip} based on which fingers are used corresponding to *i*, *j*, *k* indexes of *n* and $n_{g}$ respectively. The conditional output of $h_{i},h_{j}$, or $h_{k}$ is:

-  “nu” (not used), when the same finger is not used in current node and goal node (e.g., from n=[102001] to $n_{g}=\left[15\;25\;0\;6\right]$, finger 3 is not utilized in both nodes; in this case, the output of $h_{k}$ is “nu”).
- “ar” (addition/removal), when the current node is using a finger while the goal node doesn’t and vice versa (e.g., from n=[102001] to $n_{g}=\left[15\;0\;25\;6\right]$, the position of finger-2 is at 20th contact point in *n*, while in $n_{g}$ finger 2 is not used; similarly finger 3 is not used in the *n*, while its position is at 25th contact point in $n_{g}$. In this scenario, the output of $h_{j}$ and $h_{k}$ is “ar”).
- “dp” (direct path), when the same finger is being used in both *n* and $n_{g}$, and can directly reach from *n* to $n_{g}$, by rolling only.
- “ip” (indirect path), when the same finger is being used in *n* and $n_{g}$, but can not directly reach from *n* to $n_{g}$ by rolling only.

The determination of the two last states “dp” and “ip” requires to check if the finger can directly go from *n* to $n_{g}$ by rolling only. In that way, we compute:

(14) $$\begin{array}{cc} p_{i} & =abs(i+l-i_{g}-l_{g}), \end{array}$$

(15) $$\begin{array}{cc} p_{j} & =abs(j+l-j_{g}-l_{g}), \end{array}$$

(16) $$\begin{array}{cc} p_{k} & =abs(k+l-k_{g}-l_{g})\;. \end{array}$$

When $p_{i}=0$, $p_{j}=0$, or $p_{k}=0$, it indicates respectively that the finger 1, 2 or 3 can move from current node to goal node directly by rolling. In such a case, the output of $h_{i},h_{j}$ or $h_{k}$ is respectively “dp” (direct path). Otherwise, if the value is $p_{i}\neq 0$, $p_{j}\neq 0$, or $p_{k}\neq 0$ it will need a reconfiguration; thus, the output of $h_{i},h_{j}$ or $h_{k}$ will be “ip” (indirect path).

### 4.3. Calculation of Estimated Reconfigurations $t_{r}$

To estimate the total number of reconfigurations, we compute the minimum reconfiguration operations $t_{r}$ that are required to move from current node (*n*) to goal node ($n_{g}$). The Table 1 defines this value $t_{r}$ for every possible cases considering a 2 fingers final grasp.

The examples described in Table 1 enable to illustrate the general principle. Let us take the first data (a), i.e., n=[64301], and $n_{g}=\left[1\;38\;0\;5\right]$; for both used fingers (1 and 2), the output is “ip”, and the third finger is not used in both current and final nodes (“nu” case). We are going to show that this case requires at least 6 reconfiguration operations ($t_{r}=6$) as:

-  First, we have to add an unused finger, i.e., finger-3 to any admissible node going on to the case (b);
-  Then, remove finger-1 or finger-2, respectively, going on to case (c${}_{1}$) or (c${}_{2}$). We consider the first case in the following;
- Then, place finger-1 in such a way a direct path is possible with this finger, going on to case (d) having $h_{i}$ = “dp”;
- Then, remove finger-2, going on to the case (e);
- Place finger-2 in such a way a direct path is possible with this finger, going on to case (f) having $h_{j}$ = “dp”;
- At the end, remove finger-3 going on to the case (g) where the final node $n_{g}$ can be reached by rolling without reconfiguration.

This example illustrates the number of reconfigurations required for most of the cases described in Table 1. The last case is the case (c${}_{3}$), where the first step is to add an unused finger, i.e., finger 3 to any admissible node, going on to the case (d) already mentioned above.

For all the possible nodes (*n*), the Table 1 enables to estimate the minimum number of reconfigurations $t_{r}$ required from *n* to $n_{g}$. The cost function and heuristic function are thus completely defined.

## 5. Results

In order to illustrate the behavior and properties of the proposed method, some examples are going to be considered. These examples defined in a first subsection use several geometries and are based on physical properties of micro-objects available in literature. The first analysis will highlight the impact of adhesion and friction on the size of the number of Nodes in the Graph (*G*) in a second subsection. As in the three planar rotations defined in finger planning, the second is the most constrained one; it will be carefully analyzed in a third subsection. The two last subsections will be dedicated to the finger trajectories to perform a complete 3-D rotation of micro-objects and to the computation time enabling to show the relevance of the proposed heuristic.

### 5.1. Parameters of the Examples

**Figure 4:**
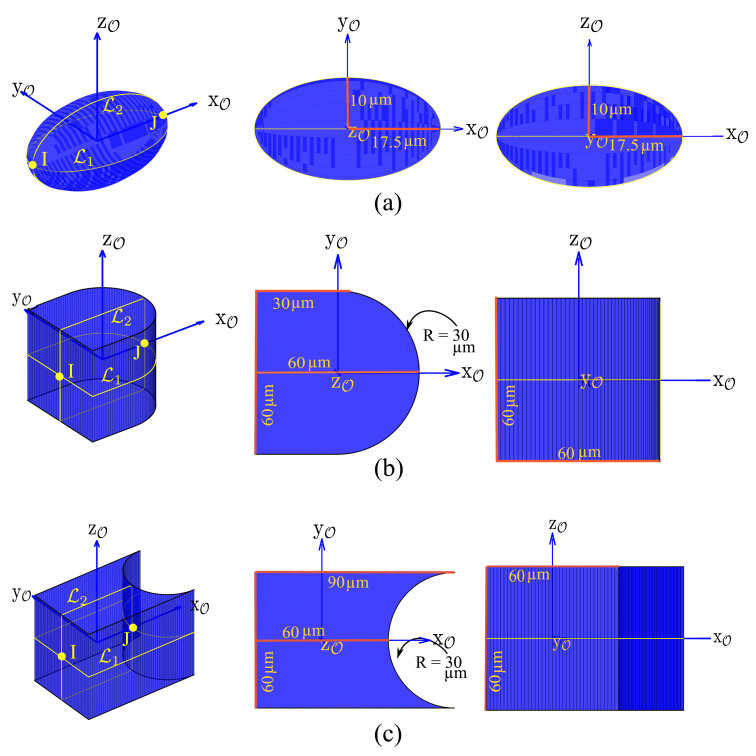
CAD model of various objects used for the simulations: (**a**) ellipsoid, (**b**) convex shaped object, (**c**) concave shaped object.

The proposed methodology presented in the previous sections has been simulated and implemented to generate the finger trajectories for three objects with different curvatures in 3-D space, i.e., *Ellipsoid*, *Convex* shaped object and *Concave* shaped object provided in Figure 4, using 3 fingers with 9 μm diameter spherical tips. For the simulations, we have considered the physical properties of Silicon for all the fingers and the objects, a pull-off force of 1.5 μN [26], and a maximal grasping force of 30 μN. As described in Section 4, we propose to decompose the movement in 3 successive rotations as: $R(z,\theta_{1})$ over XYplane$P_{1}$, $R(y,\theta_{2})$ over XZplane$P_{2}$, and $R(z,\theta_{3})$ over XYplane$P_{1}$, respectively. The sampling of the objects along $L_{1}$ and $L_{2}$ has been done using Equation (8) considering $\Delta \theta =20^{\circ }\;$.

### 5.2. Impact of Adhesion and Friction on Graph (*G*)

Table 2 represents the impact of friction coefficient on the grasp stability (no. of nodes) when adhesion is considered and when it is ignored. The contact points are sampled on the lines $L_{1}$ and $L_{2}$ curves that represent the intersection of the object illustrated in Figure 4 with the two considered rotation paths. The other parameters (finger radius, adhesion forces, and grasping forces) are constant.

We see that in the presence of adhesion forces, the number of nodes generated are the same for different values of friction coefficient. However, in the absence of adhesion forces, the friction coefficient plays an important role in the stability. Indeed, the higher the friction coefficient, the higher the grasping possibilities. In the rest of the paper, we are considering a friction coefficient of 0.3.

As a conclusion, taking into account the adhesion in microscale induces a graph *G* whose size is almost independent of the friction coefficient but whose size is significantly larger than without adhesion.

### 5.3. Analysis of the Second Rotation

As for rotations, the most challenging rotation to carry out is the intermediate one (2nd rotation), as the fingers’ positions need to start from and end at specific points (i.e., intersecting points *I*, *J* of orthogonal planes on the object). Due to this condition, the second rotation requires one or more reconfiguration, whereas for first and third rotations, they can directly be carried out by rolling most of the time.

**Figure 5:**
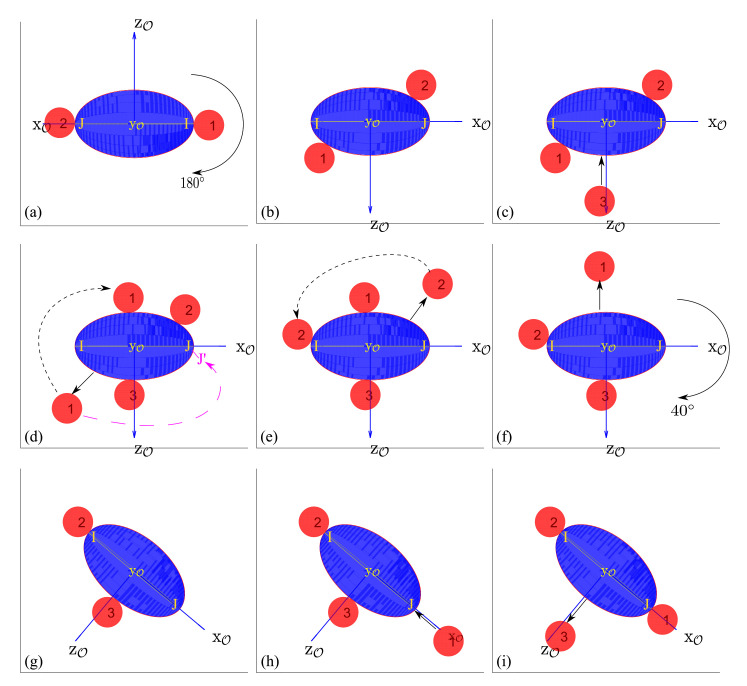
Example of a second rotation: (**a**) initial configuration, (**b**) configuration after a first object rotation of $180^{\circ }$ using finger rolling, (**c**) placement of finger-3, (**d**) moving finger-1, (**e**) moving finger-2, (**f**) removing finger-1, (**g**) finger-2 on its desired place I after object rotation of $40^{\circ }$ using finger rolling (**h**) placement of finger-1 on its desired place J, (**i**) removal of finger-3.

As mentioned, the second rotation is the most challenging to carry out because of the constraints about the initial and final positions of the fingers on the object. Figure 5 represents some steps involved in the second rotation, where the current node n=[13801] (a), and goal node $n_{g}=\left[38\;1\;0\;12\right]$ (i). It means that we expect a rotation of 11·Δθ (going from 1 to 12 in the fourth coordinates) which represents $220^{\circ }$. In the initial configuration, finger-1 is placed on I (1 as the first coordinate of *n*), finger-2 is placed on J (defined by 38 as the second coordinate of *n*), and finger-3 is not used (defined by 0 as the third coordinate of *n*). In the final configuration, finger-1 will be placed on point J, finger-2 will be placed on point I, and finger-3 will not be used.

Going from the initial configuration Figure 5a to the final configuration Figure 5i is performed in four major phases. The first phase is a half turn of the object, going from Figure 5a to Figure 5b, where the fingers are rolling on the object. The second phase is a sequence of finger gaiting in order to reach Figure 5f. In the third phase, the object reaches the final orientation Figure 5g in which finger-2 rolls to its final position I. The last phase is a second sequence of finger gaiting to place the finger-1 on point J and remove finger-3. This sequence is highly constrained by the adhesion. Indeed, in the case of 3 fingers grasping, when a finger is being removed, it pulls the object and may detach the object from the two other fingers. Concretely, only few configurations exist where a finger can be removed. This particularity of the micro-object’s behavior induces the original sequence of finger gaiting presented on Figure 5c–i.

Typically, on Figure 5d, we could expect that the finger-1 goes directly to the point J${}^{\prime }$, from where it could reach its final position by rolling. The algorithm makes another choice, because if finger-1 is directly placed on J${}^{\prime }$, then finger-2 will not be able to be removed in the next step, i.e., Figure 5e.

At the end, because of the constraint of the finger removing, the final configuration is reached after 4 finger-placements and 4 finger-removals, a total of 8 reconfiguration operations. As per the estimated reconfigurations table (Table 1), the heuristic function expects to have only 6 reconfigurational operations ($h_{i}$ is “ip”, $h_{j}$ is “ip”, and $h_{k}$ is “nu”). However, the optimal path contains 8 operations to carry out this second rotation.

As mentioned above, the second rotation starts and finishes with a two fingers grasp. Concretely, there are 6 combinations due to fingers’ permutation (${}^{3}$P${}_{2}$ = 6) which have to be evaluated. We compute all the six trajectories for second rotation and choose the optimal one. Table 3 provides an example of the cost and time computation obtained on the six possible trajectories for the second rotation. It shows that each of 6 combinations provides different number of executed reconfigurations $t_{r}^{exe}$ and a different computation time.

As we can see in Table 3, the higher the number of required reconfigurations, the higher the global cost and the computation time. In this example, the fourth case, i.e., n=[13801] and $n_{g}=\left[38\;1\;0\;12\right]$, which has the lowest cost, will be retained for the simulation purpose. This case is described in detail in Figure 5.

### 5.4. Analysis of the Whole 3-D Rotation

Our goal is to achieve 3-D manipulation of a micro-object. To manipulate a micro-object in 3-D, we proposed to decompose the 3-D rotation into three 2-D rotations and then combine them through *Euler’s Angles* as described in Section 3.1. All three individual rotations have been carried out in their object frame O; thus, for all the three individual rotations, the transformation matrix from 3rd rotation to world frame W i.e., ${}^{W}T_{3}$ is

(17) $$\begin{array}{cc} {}^{W}T_{3} & ={}^{W}T_{O}\cdot {}^{O}T_{1}\cdot {}^{1}T_{2}\cdot {}^{2}T_{3}. \end{array}$$

**Figure 6:**
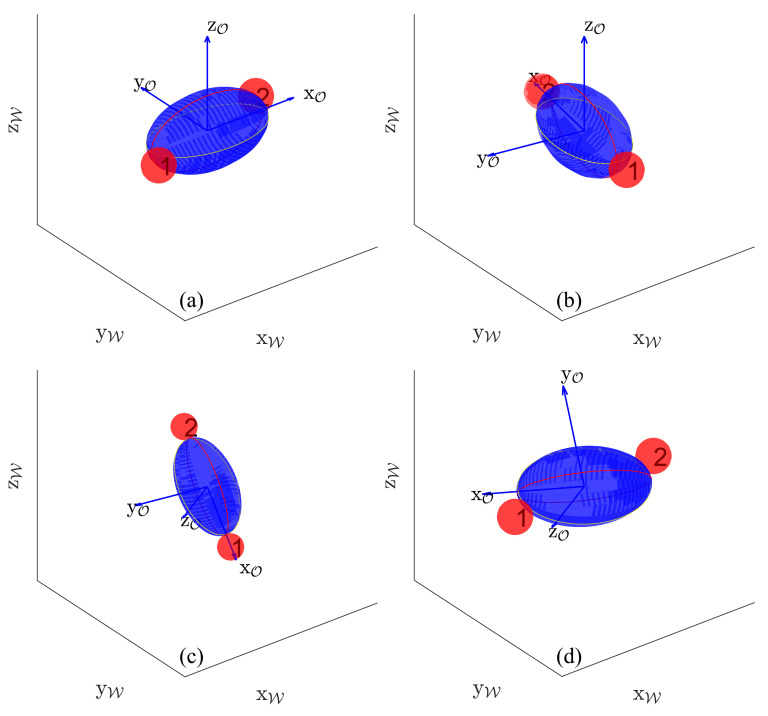
Representation of combined rotations: (**a**) initialization, (**b**) first rotation, (**c**) second rotation, (**d**) third rotation. The first and third rotations are obtained using rolling only without finger gaiting; the trajectory of the second rotation is detailed in Figure 5.

Figure 6 shows the complete 3-D rotation of an ellipsoid object. Figure 6a,b represent the initial grasp of the object by finger-1 and finger-2, and its rotation on the object’s *z*-axis of $80^{\circ }$ placing the finger-1 and finger-2 at intersecting points of $L_{1}$ and $L_{2}$. Figure 6c represents the second rotation of $220^{\circ }$ on the object’s *y*-axis initializing from desired fingers grasp (intersecting points of $L_{1}$ and $L_{2}$) encompassing all the reconfigurational steps (some steps represented in Figure 5) to reach another desired fingers grasp with finger-1 and finger-2. Figure 6d represents the last rotation of $100^{\circ }$ on the object’s *z*-axis starting from desired finger grasp. It is to be noted that in this example (ellipsoid object), the first and third rotations are executed directly by rolling (without any reconfiguration). We have also provided the Supplementary Video S1 of combined 3-D rotation, the link can be found at the end of paper in Supplementary Materials section.

### 5.5. Computation Time

The last performance analyzed in this paper is the computation time which is one way to evaluate the relevance of the proposed heuristic. Table 4 presents the computation time for three examples using the three objects’ geometries described in Figure 4 and considering both cases with or without adhesion. The ellipsoid case shows an example where the required rotations can be reached with adhesion and cannot be reached without adhesion. In such a case, the friction coefficient 0.3 is not sufficiently large to provide a sufficient number of stable grasps to connect the initial node and final node in the Graph(G). It means that it is not physically possible to find a succession of stable grasps to perform the rotations. In the two other cases, the total computation times of both cases of adhesion have a similar order of magnitude (5 to 10.5 s). This example describes a global trend regarding the impact of adhesion on the number of reconfigurations. As presented in the two last examples, in most of the cases, the number of reconfigurations is higher without adhesion than with adhesion. Indeed, without adhesion, the reachable rotation with a grasping is limited by the friction cone and possible collision between fingers, when with adhesion the rotation is only limited by the second constraint.

## 6. Discussion and Conclusions

### 6.1. Discussions

Regarding the computation time, the current results were simulated using MATLAB ^®^ with core programming, where the specifics of systems are: Intel Core i7-9750H (6 core CPU), 24-GB RAM. The computation time is also impacted by the sampling strategy defined by the parameter Δθ. A smaller Δθ will enable to consider more values of rotations but will increase the number of Nodes and thus the computation time.

In the simulations of “with adhesion cases”, we consider a constant value of pull-off force representing the force required to remove a finger from the object. Concretely, this force is highly dependent on the surface properties of both the object and the finger (local oxidation, local roughness, etc.) and may vary along the trajectory. We can show that until the pull-off force is significantly larger than the weight, the Graph(G) and thus the optimal trajectory are independent of the exact value of the pull-off force. Thus, the obtained trajectory described in this paper is robust to pull-off force variation along the trajectory.

The current simulation takes into account the adhesion phenomena, the friction force, the object geometry and the collision between the fingers considered as spheres. Concretely, the fingers should be placed on supports linked to translation micro-actuators. The optimal shape of the supports in order to reduce their collisions and the impact of these potential collisions on the finger trajectories will be studied in future works.

### 6.2. Conclusions

In this paper, we proposed a new method to perform finger path planning for 3-D dexterous manipulation of micro-objects. To leverage the complexity of 3-D dexterous manipulation, we proposed to carry out three planar rotations about two orthogonal axes. We also proposed an algorithm to cope with the constraints to starting and/or ending the rotation with predefined finger positions on the object and to ensure the continuity of the manipulation process over three successive angles. Moreover, we formalized the sampling strategy to sample contact points on the object which takes into account the finger radius, object geometry, and the rotational step. The simulation results show that the desired 3-D manipulation of micro-objects can be carried out by performing three 2-D rotations with large rotation angles. Currently, the time to compute fingers’ trajectories for first and third rotations is a few seconds, while it is a few minutes for the second rotation, which is in acceptable range for various applications. We also conclude that the presence of adhesion forces enables more feasible trajectories in contrast to the absence of adhesion forces, as the number of equilibrium grasp is higher.

## Figures and Tables

**Table 1:** Estimated number of reconfiguration $t_{r}$ in function of the non-ordered set $\left\{h_{i},h_{j},h_{k}\right\}$ for a path $n⟶n_{g}$, considering a 2-fingered grasp $n_{g}$ and some examples considering the final node $n_{g}\;=\;\left[1\;38\;0\;5\right]$.

$t_{r}$ Calculation Table			Examples: *n*⟶$n_{g}=$ [1 38 0 5]				Ref.
$\left\{h_{i},h_{j},h_{k}\right\}$	$t_{r}$	$F_{used}$	*n*	$h_{i}$	$h_{j}$	$h_{k}$	
{ip,ip,nu}	6	2	11 63 0 1	ip	ip	nu	(a)
{ip,ip,ar}	5	3	11 63 25 1	ip	ip	ar	(b)
{ip,ar,ar}	4	2	0 63 25 1	ip	ar	ar	(c${}_{1}$)
			11 0 25 1	ar	ip	ar	(c${}_{2}$)
{dp,ip,nu}	4	2	5 63 0 1	dp	ip	nu	(c${}_{3}$)
{dp,ip,ar}	3	3	5 63 25 1	dp	ip	ar	(d)
{dp,ar,ar}	2	2	5 0 25 1	dp	ar	ar	(e)
{dp,dp,ar}	1	3	5 42 25 1	dp	dp	ar	(f)
{dp,dp,nu}	0	2	5 42 0 1	dp	dp	nu	(g)

**Table 2:** Impact of friction coefficient (μ: 0.1–0.5) on the number of admissible stable grasps (no. of nodes).

Object		Number of Nodes (in Millions)						
		With Adhesion	Without Adhesion			Ratio:		
		(nwa)	(nwoa)			nwoa/nwa		
		μ∈ [0.1; 0.5]	0.1	0.3	0.5	0.1	0.3	0.5
Ellipsoid	$L_{1}$;$L_{2}$	3.373	0.6325	1.527	2.020	19%	45%	62%
Convex	$L_{1}$	40.65	12.35	21.14	26.32	30%	52%	65%
Concave	$L_{1}$	134.4	16.96	31.25	43.68	13%	23%	33%
Convex Concave	$L_{2}$	42.72	8.409	18.0	25.97	20%	42%	61%

**Table 3:** Comparison of cost and time computations for Ellipsoid’s second rotation. ${}^{‡}$ represents the time to generate path with “Tie Breaking” strategy. $t_{r}^{exe}$ is the number of reconfiguration in the optimal path.

*n*	$n_{g}$	f(n)	$\left\{h_{i},h_{j},h_{k}\right\}$	$t_{r}^{\mathrm{exe}}$	Time (s)	Time ${}^{‡}$ (s)
1 38 0 1	1 0 38 12	2240	{ip, ar, ar}	10	45	10
	0 1 38 12	2240	{ar, ip, ar}	10	50	8
	38 0 1 12	2240	{ip, ar, ar}	10	78	35
	38 1 0 12	1880	{ip, ip, nu}	8	3	2
	0 38 1 12	2240	{ar, ip, ip}	10	48	20
	1 38 0 12	2600	{ip, ip, nu}	12	857	203

**Table 4:** Comparison of the computation time to generate the finger trajectories for the objects presented in Figure 4; where $\theta_{i}$ represents the *i*th rotation to be carried out for $\theta^{\circ }$, and “NPA” represents that No Path Available.

Object and		With Adhesion				Without Adhesion			
Rotations		$t_{r}^{\mathrm{exe}}$	Time	Nodes		$t_{r}^{\mathrm{exe}}$	Time	Nodes	
$\theta_{1},\theta_{2},\theta_{3}\left(\deg.\right)$			(s)	To Be Visi.	Visited		(s)	To Be Visi.	Visited
Ellipsoid	80	0	0.38	12474	5	NPA	34207	654859	654859
	220	8	1.1	4291	1202	NPA	0.09	164	164
	100	0	0.01	206	6	NPA	0.08	164	164
Convex	60	0	4.96	41829	4	0	0.13	2661	4
	80	6	0.22	1469	25	8	8.80	30253	1211
	40	0	0.17	2246	106	2	0.17	2123	121
Concave	100	0	7.13	60254	6	2	0.29	3836	25
	60	4	2.85	5586	101	8	6.62	25873	1122
	20	0	0.27	3359	166	2	0.12	1046	162

## Supplementary Materials

The following are available online at https://www.mdpi.com/article/10.3390/mi12080986/s1. Video S1: Example of 3-D dexterous manipulation for ellipsoid micro-object.

## References

[1] Fearing R.S. Survey of sticking effects for micro parts handling. Proceedings of the 1995 IEEE/RSJ International Conference on Intelligent Robots and Systems, Human Robot Interaction and Cooperative Robots.

[2] Ozawa R., Tahara K. (2017). Grasp and dexterous manipulation of multi-fingered robotic hands: A review from a control view point. Adv. Robot..

[3] Huang Y., Liang Z., Alsoraya M., Guo J., Fan D. (2020). Light Gated Manipulation of Micro/Nanoparticles in Electric Fields. Adv. Intell. Syst..

[4] Curiotto S., Cheynis F., Muller P., Leroy F. (2020). 2D Manipulation of Nanoobjects by Perpendicular Electric Fields: Implications for Nanofabrication. ACS Appl. Nano Mater..

[5] Rauch J.Y., Lehmann O., Rougeot P., Abadie J., Agnus J., Suarez M.A. (2018). Smallest microhouse in the world, assembled on the facet of an optical fiber by origami and welded in the *μ*Robotex nanofactory. J. Vac. Sci. Technol. A Vacuum Surf. Film..

[6] Mehrabi H., Hamedi M., Aminzahed I. (2020). A novel design and fabrication of a micro-gripper for manipulation of micro-scale parts actuated by a bending piezoelectric. Microsyst. Technol..

[7] Dong L., Arai F., Fukuda T. 3D nanorobotic manipulation of nano-order objects inside SEM. MHS2000. Proceedings of the 2000 International Symposium on Micromechatronics and Human Science (Cat. No. 00TH8530).

[8] Vittoria S., Lahlou G., Torres R., Daoudi H., Mosnier I., Mazalaigue S., Ferrary E., Nguyen Y., Sterkers O. (2021). Robot-based assistance in middle ear surgery and cochlear implantation: First clinical report. Eur. Arch. Oto-Rhino.

[9] Cheng X., Huang E., Hou Y., Mason M.T. (2021). Contact Mode Guided Motion Planning for Dexterous Manipulation. arXiv.

[10] Yoneda T., Schaff C., Maeda T., Walter M. (2021). Grasp and Motion Planning for Dexterous Manipulation for the Real Robot Challenge. arXiv.

[11] Garcia-Rodriguez R., Parra-Vega V. (2021). In-hand manipulation of a circular dynamic object by soft fingertips without angle measurement. Sci. China Inf. Sci..

[12] Bullock I.M., Ma R.R., Dollar A.M. (2013). A hand-centric classification of human and robot dexterous manipulation. IEEE Trans. Haptics.

[13] Nakamura A., Nagata K., Harada K., Yamanobe N. (2017). Using simplified geometric models in skill-based manipulation for objects used in daily life. Artif. Intell. Res..

[14] Ma R.R., Dollar A.M. On dexterity and dexterous manipulation. Proceedings of the 2011 15th International Conference on Advanced Robotics (ICAR).

[15] Andrychowicz O.M., Baker B., Chociej M., Jozefowicz R., McGrew B., Pachocki J., Petron A., Plappert M., Powell G., Ray A. (2020). Learning dexterous in-hand manipulation. Int. J. Robot. Res..

[16] Seon J.A., Dahmouche R., Gauthier M. (2017). Enhance in-hand dexterous micromanipulation by exploiting adhesion forces. IEEE Trans. Robot..

[17] Cherfia A., Zaatri A., Giordano M. (2020). Kinematics Analysis of a Parallel Robot with 3 DOF and 4 Segments in Pure Translation. (Dept. M). MEJ. Mansoura Eng. J..

[18] Wason J.D., Wen J.T., Gorman J.J., Dagalakis N.G. (2012). Automated multiprobe microassembly using vision feedback. IEEE Trans. Robot..

[19] Agnus J., Chaillet N., Clévy C., Dembélé S., Gauthier M., Haddab Y., Laurent G., Lutz P., Piat N., Rabenorosoa K. (2013). Robotic microassembly and micromanipulation at FEMTO-ST. J. Micro-Bio Robot..

[20] Wason J.D., Wen J.T., Dagalakis N.G. Dextrous manipulation of a micropart with multiple compliant probes through visual force feedback. Proceedings of the 2011 IEEE International Conference on Robotics and Automation (ICRA).

[21] Brazey B., Dahmouche R., Seon J.A., Gauthier M. (2016). Experimental validation of in-hand planar orientation and translation in microscale. Intell. Serv. Robot..

[22] Liseli J.B., Dahmouche R., Kumar P., Seon J.A., Gauthier M. Enhancing in-hand dexterous micro-manipulation for real-time applications. Proceedings of the 2018 IEEE 14th International Conference on Automation Science and Engineering (CASE).

[23] León B., Morales A., Sancho-Bru J. (2014). From Robot to Human Grasping Simulation.

[24] Adam G., Chidambaram S., Reddy S.S., Ramani K., Cappelleri D.J. (2021). Towards a Comprehensive and Robust Micromanipulation System with Force-Sensing and VR Capabilities. Micromachines.

[25] Euler L. (1770). Problema algebraicum ob affectiones prorsus singulares memorabile. Novi Commentarii Academlae Scientiarum Petropolitanae.

[26] Gauthier M., Alvo S., Dejeu J., Tamadazte B., Rougeot P., Régnier S. (2013). Analysis and specificities of adhesive forces between microscale and nanoscale. IEEE Trans. Autom. Sci. Eng..

